# Multiple secondary outcome analyses: precise interpretation is important

**DOI:** 10.1186/s13063-021-05975-2

**Published:** 2022-01-10

**Authors:** Richard A. Parker, Christopher J. Weir

**Affiliations:** Edinburgh Clinical Trials Unit, Usher Institute, University of Edinburgh, Edinburgh, UK

**Keywords:** Multiple testing, Secondary outcomes, Family-wise error rate, Per-comparison wise error rate, Type-I error, Multiplicity

## Abstract

Analysis of multiple secondary outcomes in a clinical trial leads to an increased probability of at least one false significant result among all secondary outcomes studied. In this paper, we question the notion that that if no multiplicity adjustment has been applied to multiple secondary outcome analyses in a clinical trial, then they must necessarily be regarded as exploratory. Instead, we argue that if individual secondary outcome results are interpreted carefully and precisely, there is no need to downgrade our interpretation to exploratory. This is because the probability of a false significant result for each comparison, the per-comparison wise error rate, does not increase with multiple testing. Strong effects on secondary outcomes should always be taken seriously and must not be dismissed purely on the basis of multiplicity concerns.

## Background

Clinical trials commonly designate one or more outcomes as primary and the rest as secondary or exploratory. Multiple testing of secondary outcomes leads to an increase in the family-wise error rate (FWER): the probability of at least one false significant result (type I error) among all secondary outcomes studied. This is why research studies often adjust for multiplicity when performing secondary outcome analyses. However, this is not always necessary. In particular, if all secondary outcome results are required to be statistically significant to conclude an overall treatment effect, then the type I error at the level of the individual tests is not relevant, and multiple testing adjustment is not appropriate [1, 2].

Multiple testing adjustment is usually achieved using a multiple testing correction, such as Bonferroni, to achieve overall type I error rate control [3–6]; but there also appears to be a growing trend for pre-specified hierarchical testing structures, whereby the secondary outcomes are tested in a pre-specified order [7–9] or by using a more complex graphical adjustment scheme [10–12]. Alternatively, researchers frequently adopt an exploratory interpretation. For example, in a clinical trial by Cao et al. [13] of a COVID-19 treatment conducted in 2020, no correction for multiplicity was applied, and the authors indicated that “the [confidence] intervals should not be used to infer definitive treatment effects for secondary outcomes” [13]. Another example is the RICH trial publication [14], where the authors write “Statistical analyses of secondary end points were not adjusted for multiplicity. Therefore, because of the potential for type I error, findings should be interpreted as exploratory.” [14] Both of these examples are consistent with the widely reported belief that if no multiplicity adjustment has been applied to multiple secondary outcomes in a clinical trial, then they must necessarily be regarded as exploratory [15–17]. In this article, we outline some counter-arguments to this view before suggesting a careful and valid way to interpret multiple secondary outcomes that neither requires an exploratory interpretation of secondary outcomes nor requires formal multiple testing adjustment.

### Issues with the “exploratory interpretation requirement” view

One interpretational issue with necessitating an exploratory interpretation of secondary outcomes in the absence of multiplicity adjustment is that pre-defined secondary endpoints will be classified in the same category as outcomes of lower importance in a trial, noting that many trials will pre-specify a list of secondary endpoints and a separate list of exploratory (or tertiary) endpoints. By considering all non-primary outcomes as exploratory, this would preclude the need to distinguish between the two. Such an approach, however, fails to recognize the many situations where secondary outcomes are critical to the overall interpretation of a clinical trial, for example in the case where the individual components of a composite primary outcome are included among the secondary outcomes. Secondary endpoint results will often feed directly into the overall interpretation of a clinical trial and facilitate understanding of the scope of any potential intervention effect. In contrast, tertiary endpoints are likely to be more exploratory, novel, hypothesis-generating, or mechanistic. For example, tertiary endpoints could be used to explore disease processes or the mechanisms by which an intervention is effective. By themselves, these tertiary endpoints are unlikely to modify the key conclusions in a clinical trial, but they may still be of interest for guiding future research. It is therefore important to retain the distinction between the two to facilitate appropriate trial interpretation.

Another problem is that if a secondary variable shows a clinically important effect or is highly significant (for example, with *P* < 0.0001), as one of the secondary outcomes in Baggot et al. [17] was found to be, does this mean that it is *only* permissible to interpret this result as exploratory if no adjustment has been made for multiple testing? Based on Gao et al., the probability of a false positive conclusion (type I error) conditional on this *p*-value will be no greater than 0.018 [18]. Therefore, given that we will have confidence that such a significant result is real and replicable, is it really fair to downgrade this finding to “exploratory”? Indeed, there is the danger that strong adverse effects on safety outcomes may not be taken seriously if they are among multiple outcomes.

To highlight the reality of this danger, consider the large COMPASS trial reported in 2019 [19], which randomized over 17,000 patients to receive proton pump inhibitors (PPIs) [19]. The trial authors reported a statistically significant increased risk of enteric infections in those allocated to a PPI (odds ratio 1.33, 95% confidence interval 1.01 to 1.75) [19]. However, in their trial publication, the authors stated that the “data in the current randomized trial were not adjusted for multiple testing, so this result should be interpreted with caution,” even though “enteric infection” was a pre-specified safety outcome [19, 20]. Although caution may indeed have been merited on the basis of the relatively high *p*-value (0.04), modest odds ratio, and wide confidence interval; we would question whether any safety signals should be treated any less seriously simply because they were one among many different safety outcomes.

A third problem is that it may lead researchers and other stakeholders to implicitly link the value of secondary outcome results with the number of secondary outcomes reported, such that their value may depreciate as the number of outcomes reported increases.

### The “per-comparison-wise error rate” approach

The relationship between the number of tests performed and the overall type I error rate is well known; what is much less well appreciated is that the *probability* of a false significant result for *each* comparison of an outcome between treatment groups, the “per-comparison-wise error rate” (PCWER), does not increase with multiple testing [2, 21, 22]. That means that if we adopt a precise, focused interpretation of the individual results, then there is no need to either apply a multiplicity adjustment or downgrade our interpretation to “exploratory.” Indeed, we would argue that strong interpretations could be made if secondary outcomes are interpreted very precisely and carefully according to the specific variables used in the analysis. This means, for example, instead of vaguely indicating that the “intervention has an effect,” we more specifically state that the “intervention has a specific effect on systolic blood pressure at 12 weeks.”

Furthermore, to take the example considered earlier of a clinical trial in COVID-19, Cao et al. write: “28-day mortality was numerically lower in the lopinavir–ritonavir group than in the standard-care group” [13]. In this case, the authors are making a specific statement about treatment efficacy for the secondary outcome of mortality at 28 days. The probability of a false significant result is only inflated if the authors were claiming generic treatment efficacy from this one outcome; but since the statement is specific to the 28-day mortality outcome, then there is no need to downgrade the interpretation to “exploratory.”

Similar arguments have been made by Rubin [1], who reasons that individual testing does not require multiplicity adjustment if we are making “specific inference” relating to individual null hypotheses [1].

Precise interpretation involves interpreting outcomes in a way that refers to all of their distinguishing features (e.g., time point, type of outcome, intervention) as appropriate, so that we can differentiate clearly between underlying individual hypothesis tests.

### Advantages of the precise interpretation

Firstly, an advantage of using the precise interpretation is that it prevents pre-specified secondary outcomes, especially safety outcomes, from being undervalued or dismissed as unimportant. As noted previously, strong effects on secondary outcomes are highly likely to be real and replicable [18] and therefore must be taken seriously regardless of the number of comparisons performed.

Secondly, it promotes a careful interpretation of secondary outcomes that is not tied to the number of multiple tests performed. After all, it does not make sense for the total number of secondary outcomes to have a bearing on how the finding for each individual outcome is interpreted [23].

Thirdly, it reduces the potential for selective reporting bias, whereby in fear of the negative consequences or perceptions of multiple testing, authors only select the most interesting secondary outcome results for formal reporting [24].

### Note of caution

A note of caution is needed at this point however. If a main trial result is *not* significant, and a secondary outcome is simply the primary outcome measured at a different time point, then care is needed that this does not becomes a way of inferring an overall treatment effect by the “back door.” Success of a treatment on a secondary outcome should not in general overcome a lack of benefit found on the primary, even if that secondary outcome were pre-specified.

More generally, if a set of secondary outcomes consist of the same outcome measured at different time points, and only one of these outcomes is statistically significant, precise interpretation requires us to take great care not to conclude that an intervention is effective on the basis of a single result without reference to the findings at the other time points. Such care may also be needed if different secondary outcomes are expected to be highly correlated with each other. In this case, interpretation of a single outcome cannot be divorced from the other outcomes that are highly correlated. This is one reason why it is crucial to transparently report *all* related secondary outcome results and statistical tests within the same study report or publication and ensure that one secondary outcome result is not unduly promoted over and above other secondary outcomes that are strongly related [25].

In contrast, for *distinct* secondary outcome comparisons, it is perfectly natural to make individual interpretations for each comparison and that this is reflected by control of the PCWER rate rather than control of the FWER. As we have argued elsewhere in the context of multi-arm trials, it does not make sense to control the FWER rate in this situation when our interpretation is at the per-comparison level [21].

## Conclusions

Multiple secondary outcomes are by definition subsidiary to primary outcomes, but this does not mean that they must necessarily be downgraded to the level of exploratory in the absence of multiplicity adjustment. Indeed, if individual secondary outcome results are interpreted precisely, then the number of tests performed is irrelevant because the per-comparison-wise error rate is not increased. What we are advocating is a careful and precise interpretation of secondary outcome results. Strong effects on secondary outcomes should always be taken seriously and must not be dismissed purely on the basis of multiplicity concerns.

## Data Availability

Not applicable.

## Abbreviations

PCWER: Per comparison-wise error rate
FWER: Family-wise error rate
